# Henoch-Schönlein purpura nephritis associated with monoclonal gammopathy of renal significance: a case report

**DOI:** 10.1590/1414-431X20198222

**Published:** 2019-07-10

**Authors:** Hui Zhao, Wen-hui Huang, Jun-yue Huang, Shou-yan Lu, Ya-hong Yang, Zhi-gang Ma

**Affiliations:** Department of Nephrology, Gansu Provincial Hospital, Lanzhou, Gansu, China

**Keywords:** Monoclonal gammopathy of renal significance, Henoch-Schönlein purpura nephritis, Chemotherapy

## Abstract

Monoclonal gammopathy of renal significance (MGRS) can present with different morphologic features and lead to kidney failure. The Henoch-Schönlein purpura nephritis (HSPN) that cannot be relieved by treatment with glucocorticoid and immunosuppressive agents suggests the presence of monoclonal gammopathy in adult patients. The present study reports on a single case of HSPN associated with IgA-κMGRS. The patient who suffered from recurrent skin purpura for 6 months and nephrotic syndrome for 2 months was admitted to our hospital. Bone marrow biopsy showed monoclonal gammopathy of undetermined significance. Kidney biopsy indicated a Henoch-Schönlein purpura nephritis (HSPN, ISKDC classified as type III) with positive staining with κ-light chain in the glomeruli and renal tubular epithelial cells. Furthermore, skin biopsy showed leukocytoclastic vasculitis and negative staining for Congo red and light chain. Given both the renal and cutaneous involvement, the patient was considered to have HSPN associated with IgA-κMGRS. The patient experienced an exacerbation in his purpura-like lesions and clinical status after treatment with glucocorticoid and immunosuppressive agents. Consequently, the patient was put on a regimen that included dexamethasone (20 mg on the 1st, 4th, 8th, and 11th days of each month, *iv*) and bortezomib (2.4 mg on the 1st, 4th, 8th, and 11th days of each month, *iv*). Eight weeks after treatment, he had complete resolution of his cutaneous purpura and his biochemical parameters improved. The latent presence of MGRS in cases of HSPN should be considered in adult patients. Increased cognizance and correct treatment options could improve patient outcomes.

## Introduction

Henoch-Schönlein purpura (HSP) is a systemic vasculitis of small and medium-sized vessels. The clinical presentation of HSP involves cutaneous palpable purpura, arthralgia/arthritis, bowel angina, and hematuria/proteinuria (1). HSP is rarely seen in adults, with an estimated annual incidence of 1.3 cases per 100,000. The disease tends to be self-limited in children (2,3), while it is more severe in older patients (3,4). Skin purpura, a classical feature of HSP, can be also observed in patients with autoimmune diseases and hematological malignancies, including multiple myeloma and amyloidosis.

Monoclonal gammopathy (MG) is a result of a clonal proliferation of lymphocytes or plasma cells that involves three main components: skin, kidneys, and peripheral nervous system (nerves). MG of renal significance (MGRS) involves the kidney and can lead to kidney failure (5,6). Morbidities associated with MGRS are high due to the severe renal lesions and associated systemic alterations (7). The present study reports a single case of a patient with Henoch-Schönlein purpura nephritis associated with IgA-κMGRS that was gradually improved by dexamethasone and bortezomib chemotherapy.

## Case Report

A 61-year-old male retired teacher who suffered from skin purpura was admitted to the local hospital. The patient was diagnosed with HSP and received oral prednisone (30 mg/day) in July 2016. Approximately one week later, the purpura disappeared, but it recurred after 20 days. Full blood examination appeared normal, as well as serum creatinine, serum calcium, liver function, and serum immunoglobulin (Ig) levels. The patient's 24-h urine protein was 0.352 g/day, which was above the reference range of 0.00–0.14 g/day). The patient was then prescribed prednisone (30 mg/day) combined with angiotensin receptor antagonist. Ten days later, his purpura disappeared, and the prednisone dose was gradually reduced to 15 mg daily. While on prednisone, his purpura recurred intermittently and was therefore was admitted to Gansu Provincial Hospital in July 2017 for further testing. He initially declined a renal biopsy, and was prescribed an increased dose of oral prednisone (40 mg/day), which was not effective. Two months later, 24-h urine protein increased to 3.399 g/day. His treatment was altered to a reduced dose of prednisone (15 mg/day) combined with cyclosporin A (100 mg, bid). In October 2017, the proteinuria improved (24-h urine protein decreased to 0.828 g/day), but he developed a purpuric rash on his extremities and torso. Consequently, he was hospitalized with purpuric skin lesions and edema on his lower limbs.

Urine analysis showed microscopic hematuria; serum albumin was decreased to 37.5 g/L (reference range 40–55 g/L) with an otherwise normal liver. Erythrocyte sedimentation rate was elevated (29 mm/h, reference range 0–15 mm/h). Serological tests for hepatitis B, hepatitis C, and human immunodeficiency virus, as well as cryoglobulin, cold agglutinin, and C3 and C4 were all negative. Serum immunoglobulin (Ig) levels showed elevated IgA (6.35 g/L, reference range 0.82–4.53 g/L), but decreased IgG (6.44 g/L, reference range 7.51–15.6 g/L) and IgM (0.40 g/L, reference range 0.46–3.04 g/L). Serum free light chains showed normal κ light chain levels (1.77 g/L, reference range 1.70–3.70 g/L) and decreased λ light chain (0.71 g/L, reference range 0.90–2.10 g/L), with a normal κ/λ ratio of 2.49 (reference range 1.47–2.95). Serum immunofixation electrophoresis pattern showed that there was an M protein band on the electrophoretic pattern (ELP), which formed a specific reaction precipitation zone with anti-IgA and anti-κ chain. In addition, urine light chain analysis revealed Bence-Jones proteinuria with λ chain level of 0.04 g/L (reference range 0 g/L) and κ chain level of 0.11 g/L (reference range 0 g/L). The graph of urine Bence-Jones protein electrophoresis also showed that there was an M protein band on the ELP. Investigations for autoimmune and infective causes were negative. Echocardiography and X-ray of the chest, skull, and pelvis were normal. Bone marrow biopsy revealed increased plasma cells accounting for 0.67% of the marrow cellularity.

## Kidney biopsy

**Figure 1.**
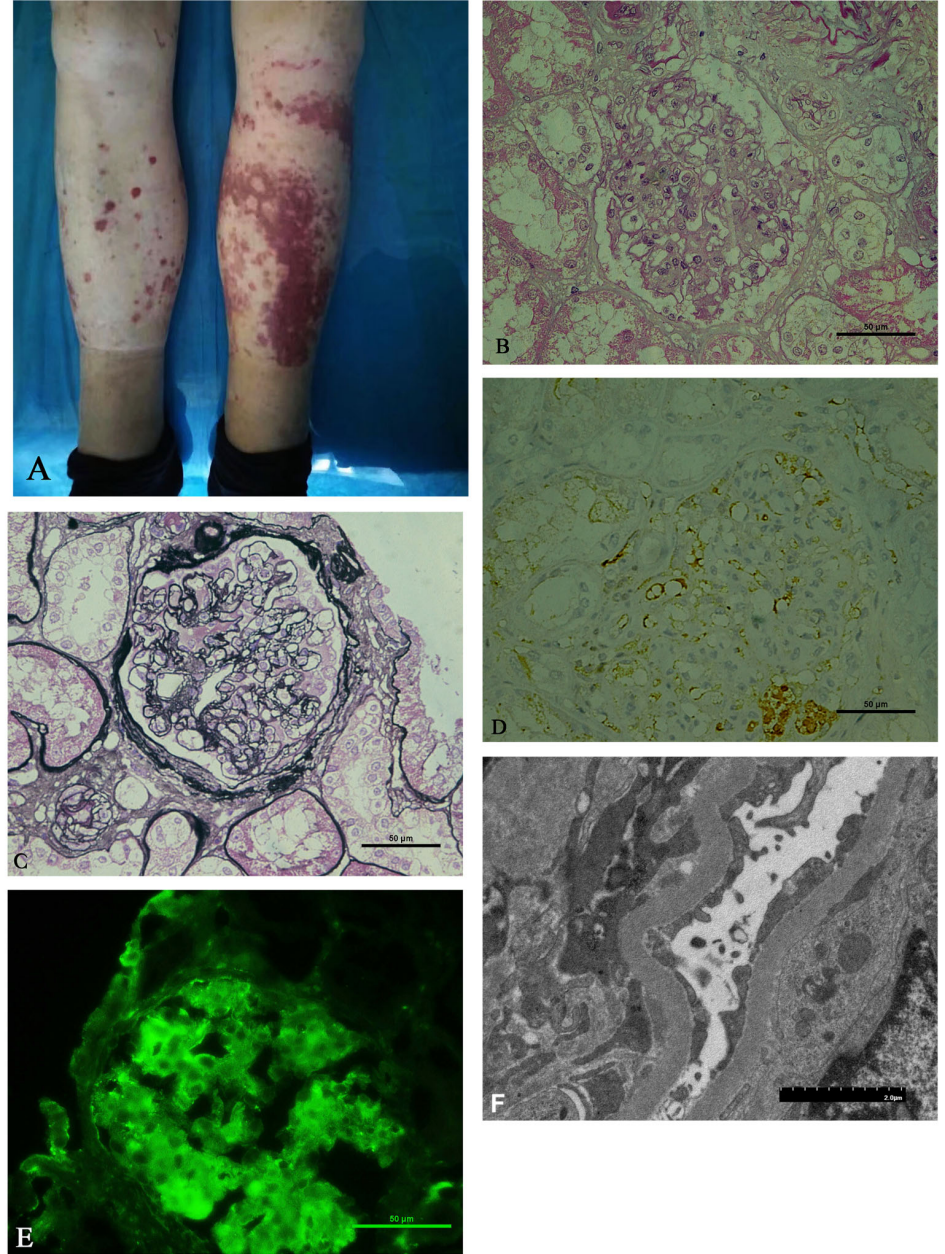
Skin purpura and kidney biopsy. **A**, Lower extremity purpura. **B**, Hematoxylin and eosin stain showing glomerular necrosis (×400). **C**, Periodic Schiff-Methenamine stain showing proliferation of mesangial cell and mesangial matrix (×400). **D**, Positive staining with κ-light chain in the glomeruli and renal tubular epithelial cells (×400). **E**, Confluent, granular staining of mesangial IgA deposits (×400). **F**, Electron microscopy demonstrating the electron-dense deposits in the mesangial region (×5000). Scale bars in **B**, **C**, **D**, **E**: 50 μm; scale bar in **F**: 2 μm.

The patient underwent a kidney biopsy in November 2017 that revealed HSP nephritis (HSPN, ISKD classified as type III, defined as focal hyperplasia and sclerosis), with isolated positive κ-light chain and negative lambda light chain immunostaining (Figure 1). Light microscopy indicated a proliferation of mesangial cells and mesangial matrix, with only a minority of tissue samples showing segmental sclerosis. Segments of some glomeruli showed the mesangial matrix expanding into the endothelial cells. Congo red staining for amyloid and IgG subclass was negative. Moderate confluent granular staining of IgA deposits was identified in the mesangium. Renal interstitial fibrosis was <10%. Electron microscopy demonstrated electron-dense deposits in the mesangial region.

## Skin biopsy

**Figure 2.**
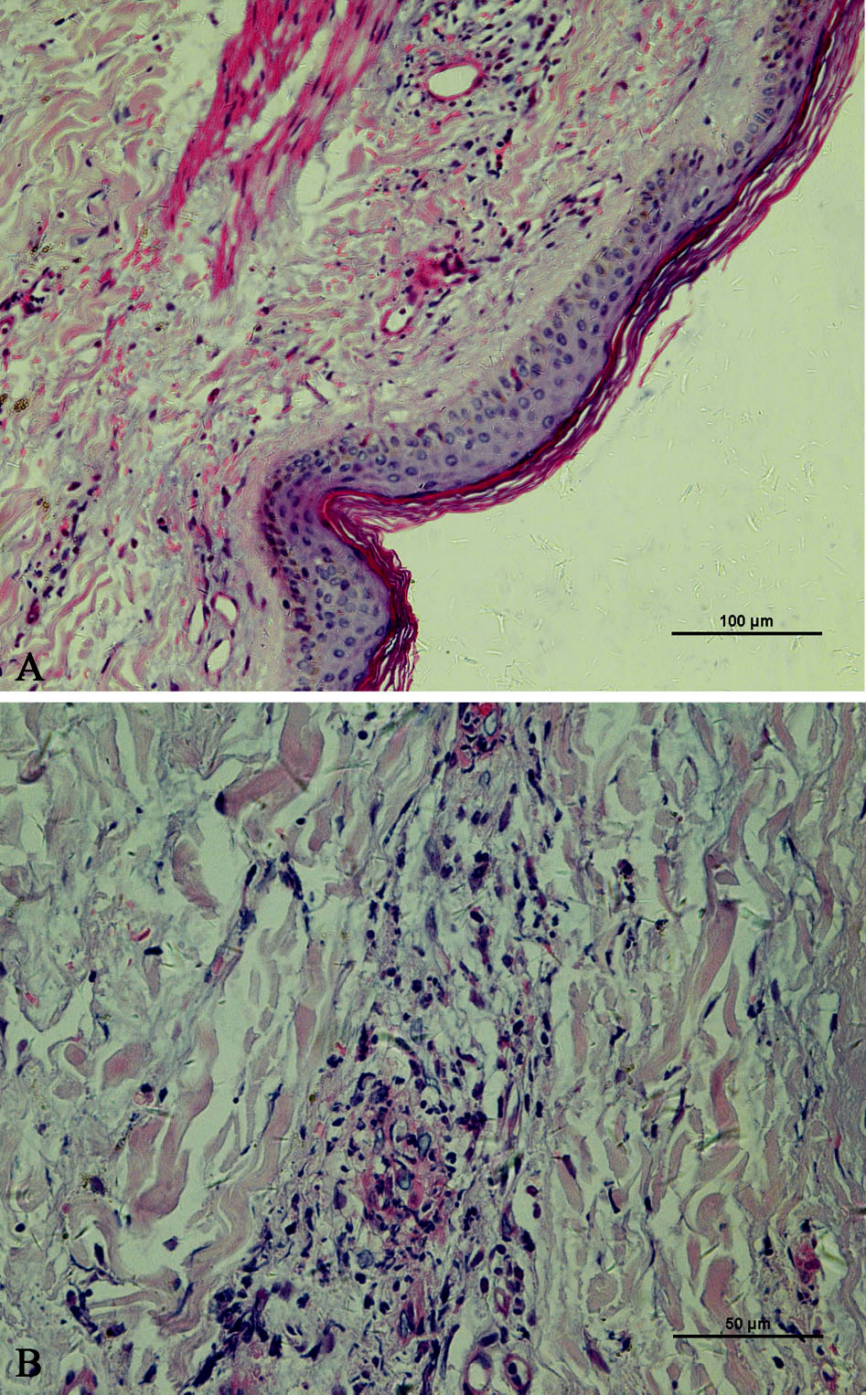
Hematoxylin and eosin stain showing chronic inflammatory cell infiltration around small blood vessels (**A**, ×200) and fibrous connective tissue hyperplasia (**B**, ×400). Scale bars: 100 μm (**A**) and 50 μm (**B**).

In November 2017, the patient underwent skin biopsy that showed leukocytoclastic vasculitis and negative staining for Congo red and κ-light chain (Figure 2).

## Clinical follow-up

The patient received intravenous methylprednisolone (320 mg/day) for seven days, and intravenous cyclophosphamide (600 mg/week) combined with intravenous dexamethasone (10 mg/week) for the next three weeks. Nevertheless, no improvement in purpura was observed one month after therapy. In addition, his serum creatinine levels increased to 2.85 mg/dL (reference range, 0.59–1.20 mg/dL); hemoglobin decreased to 62 mg/dL (reference range, 120–160 mg/dL), and proteinuria (24-h urine protein, 5.4 g/day) did not improve. One month later, the patient presented with worsened edema and lethargy. His serum creatinine further increased to 3.08 mg/dL while his serum albumin decreased to 22.7 g/L. Considering that his condition continued to deteriorate, the patient was placed on our recommended regimen that included dexamethasone (20 mg on the 1st, 4th, 8th, and 11th days of each month, *iv*) and bortezomib (2.4 mg on the 1st, 4th, 8th, and 11th days of each month, *iv*). Eight weeks after treatment, the patient had complete resolution of the cutaneous purpura, edema, and lethargy. His biochemical parameters also improved, and his serum creatinine decreased to 1.00 mg/dL, serum albumin increased to 39.9 g/L, and 24-h urine protein decreased to 1.9 g/24 h. His serum κ light chain and IgA level returned to normal. Following six courses of chemotherapy, the skin lesions were markedly improved. In August 2018, follow-up examination showed that the serum k light chain and the serum creatinine were normal, serum albumin increased to 42.1g/L, hemoglobin increased to 110 mg/dL, and 24-h urine protein decreased to 0.168 g/day. He has had no further relapses since starting this regime and there has been no further significant proteinuria or hematuria.

## Diagnosis

Based on the above results, the patient was diagnosed with HSPN associated with IgA-κ MGRS.

## Discussion

In children, HSP is usually self-limiting, while severe cases are more commonly seen in adults, and it usually responds to steroids (3). An intriguing aspect of this case involved the potential relationship between the patient's refractory HSP and a hematological malignancy. Recurrent purpura and nephritis may occur in paraproteinemias (8). Skin purpura can also occur in hematological malignancies such as multiple myeloma (MM). Cutaneous involvement associated with MM varies from 5 to 10% (9). Our patient did not meet the diagnostic criteria for MM. In addition, cryoglobulinemia can induce a thrombotic vasculopathy, and the first clinical lesion is usually a stellar or retiform purpura, which evolves into necrosis. It initially occurs on cold-exposed areas such as helix, tip of the nose, fingers, and toes (10). Our patient's cryoglobulin was negative. However, the skin biopsy revealed leukocytoclastic vasculitis and negative staining for Congo red and κ-light chain, without IgA deposition. There was no direct evidence of skin purpura-like lesions with monoclonal gammopathy and IgA vasculitis. Purpuric skin lesions in our patient spread to his extremities 1 year before the current presentation. Peculiarly, the patient experienced an improvement in skin purpura and proteinuria at the beginning of treatment with glucocorticoid, followed by an exacerbation in his purpura-like lesions and clinical status after treatment with glucocorticoid and immunosuppressive agents. Given the clinical severity, the patient was treated with dexamethasone and bortezomib. After eight weeks of treatment, he had a complete resolution of his cutaneous purpura and biochemical parameters. Therefore, HSPN associated with IgA-κ MGRS was thought to be responsible for purpuric skin lesions in this patient. Accordingly, our patient was diagnosed with HSPN with IgA-κMGRS upon hospitalization.

In 2012, the term MGRS was introduced to distinguish monoclonal gammopathies that result in the development of kidney disease from those that are benign. MGRS patients have a small B-cell clone and a low level of circulating paraprotein (11). In April 2017, the International Kidney and Monoclonal Gammopathy Research Group (IKMG) redefined MGRS as a clonal proliferative disorder that produces a nephrotoxic monoclonal immunoglobulin and does not meet previously defined hematological criteria for treatment of a specific malignancy (12). Renal damage is caused by the deposition of secreted monoclonal immunoglobulin (MIg) or its fragments, produced by a B-cell or plasma cell clone. Kidney biopsy is therefore required to determine the exact nature of the lesion and severity of renal disease, and immunofluorescence and electron microscopy studies are often required to determine the clonality and the deposit pattern (13).

In MGRS cases, tumor burden is not high and the determination of the latent renal injury is based on the physicochemical property of the paraprotein (5). Monoclonal immunoglobulin-related diseases tend to be progressive and are unlikely to undergo spontaneous remission (14,15). MGRS is associated with high morbidity, and treatment is usually necessary to prevent deterioration of renal function. In most cases, overall survival of patients with MGRS is significantly better than that of patients with multiple myeloma, but the renal outcomes are not as good (16). In the most recent observational studies, nine out of 2935 monoclonal gammopathy of undetermined significance (MGUS) patients (approximately 1.5%) were diagnosed with MGRS. Among MGRS patients, the incidence of progression to MM was significantly higher than in MGUS patients (18 *vs* 3%) (17).

To date, there is no available strategy that could inhibit or clear the monoclonal immunoglobulin tissue deposition. Monoclonal diseases are poorly responsive to conventional immunosuppression and instead require clone-directed therapy (18). Therefore, targeting the underlying B-cell clone with chemotherapy, although it is not an evidently malignant clone per se, is the only available therapeutic option for MGRS (19). The choice of chemotherapeutic agents should take into account the drugs’ renal clearance and potential renal and non-renal toxicity (20). The introduction of novel agents like bortezomib has changed the course of myeloma patients on the overall and renal survival curves (21). The Greek myeloma group reviewed myeloma patients that presented with renal impairment from 1990 to 2011 and noted that overall survival improved significantly in the last 10 years with the introduction of thalidomide in 2000 and bortezomib in 2005 (22).

HSPN has been reported very occasionally in patients with IgA monoclonal gammopathy or MM (23–25). As can be seen from the treatment course of our case, IgA paraproteinemia plays an important role in nephropathy. Therapeutic choices should be determined taking into account the renal characteristics of the disease, particularly the risk of renal deterioration, the presence and severity of extrarenal manifestations, and the safety profile of chemotherapy agents in renal impairment (13).

In conclusion, HSPN that cannot be relieved by treatment with glucocorticoid and immunosuppressive agents suggests the presence of monoclonal gammopathy in adult patients. We must bear in mind the latent presence of MGRS in cases of HSPN.
